# Early Experience of the First Single-Room Gantry Mounted Active Scanning Proton Therapy System at an Integrated Cancer Center

**DOI:** 10.3389/fonc.2020.00861

**Published:** 2020-05-29

**Authors:** Matthew K. Forsthoefel, Elizabeth Ballew, Keith R. Unger, Peter H. Ahn, Sonali Rudra, Dalong Pang, Sean P. Collins, Anatoly Dritschilo, William Harter, Nitika Paudel, Brian T. Collins, Jonathan W. Lischalk

**Affiliations:** Department of Radiation Medicine, Georgetown University Hospital, Washington, DC, United States

**Keywords:** proton therapy, pencil-beam scanning, cancer, insurance coverage, Monte Carlo

## Abstract

**Introduction:** Review the early experience with a single-room gantry mounted active scanning proton therapy system.

**Material and Methods:** All patients treated with proton beam radiotherapy (PBT) were enrolled in an institutional review board-approved patient registry. Proton beam radiotherapy was delivered with a 250 MeV gantry mounted synchrocyclotron in a single-room integrated facility within the pre-existing cancer center. Demographic data, cancer diagnoses, treatment technique, and geographic patterns were obtained for all patients. Treatment plans were evaluated for mixed modality therapy. Insurance approval data was collected for all patients treated with PBT.

**Results:** A total of 132 patients were treated with PBT between March 2018 and June 2019. The most common oncologic subsites treated included the central nervous system (22%), gastrointestinal tract (20%), and genitourinary tract (20%). The most common histologies treated included prostate adenocarcinoma (19%), non-small cell lung cancer (10%), primary CNS gliomas (8%), and esophageal cancer (8%). Rationale for PBT treatment included limitation of dose to adjacent critical organs at risk (67%), reirradiation (19%), and patient comorbidities (11%). Patients received at least one x-ray fraction delivered as prescribed (36%) or less commonly due to unplanned machine downtime (34%). Concurrent systemic therapy was administered to 57 patients (43%). Twenty-six patients (20%) were initially denied insurance coverage and required peer-to-peers (65%), written appeals (12%), secondary insurance approval (12%), and comparison x-ray to proton plans (8%) for subsequent approval. Proton beam radiotherapy approval required a median of 17 days from insurance submission.

**Discussion:** Incorporation of PBT into our existing cancer center allowed for multidisciplinary oncologic treatment of a diverse population of patients. Insurance coverage for PBT presents as a significant hurdle and improvements are needed to provide more timely access to necessary oncologic care.

## Background

Proton beam radiotherapy (PBT) has distinct advantages over standard x-ray radiotherapy as a consequence of the physical superiority of the Bragg peak, which can reduce radiation doses to adjacent organs at risks (OARs) as well as the integral dose of radiation delivered to patients. Standard x-ray therapy delivers dose along the entire length of the beam path including the normal tissues both proximal and distal to the tumor (1). Exit dose leads to additional dose delivered to surrounding OARs that may increase the risk of radiation-induced acute and late toxicity (2–9). PBT has been widely utilized for decades with excellent outcomes for pediatric malignancies, including medulloblastoma, ependymoma, and rhabdomyosarcoma, among others (10–13). Reduction of normal tissue radiation exposure is particularly important for younger patients due to increased tissue sensitivity to even low doses of radiation therapy and consequently the development of long-term toxicities and secondary malignancies (14–16). Retrospective studies have shown better cognitive and neuropsychological outcomes in pediatric patients with brain and central nervous system (CNS) tumors following PBT compared to x-ray radiation therapy (17–19). PBT has also demonstrated a role in multiple adult tumors, including primary malignancies of the central nervous system (CNS), gastrointestinal (GI) tract, genitourinary (GU) tract, and thorax, among others, with promising outcomes (20–27).

In the United States, PBT was first adapted for clinical use in the 1950s at Berkeley National Laboratories followed by Massachusetts General Hospital in the 1960s (28, 29). According to the particle therapy cooperative group (PTCOG), the number of centers offering PBT since that time has expanded to 35 active proton centers across the United States by the end of 2019 with an additional 13 centers under construction or planned. Many centers are comprised of a single cyclotron that supplies the accelerated proton to several different treatment rooms, with each room dependent on the same accelerator. However, the facility required to house the accelerator and treatment rooms can be massive and are frequently located at a separate facility remote from the primary cancer care. This limits accessibility to critical services such as medical oncology, surgical oncology, anesthesiology services, and the primary radiation oncology department at the main cancer center. The extraordinary cost associated with building and maintaining these large multiroom facilities and lack of adequate space available at some centers have been major factors in limiting the availability of PBT across the country. Even once funded, these large facilities can struggle financially due to accrued debt and maintenance cost (30–32). An alternative to the multi-gantry proton facility is a single-gantry proton therapy room that may require reduced overall space, upfront overhead, and continued maintenance. Cost containment is likely one reason for the expansion of proton centers across the country (33).

Our academic institution installed a single-vault compact active scanning proton therapy system with a gantry mounted accelerator, the Mevion S250i with HyperScan and Adaptive Aperture in February 2018 (Mevion Medical Systems, Inc., Littleton, MA). In this study, we aim to detail our early institutional experience of patients treated with single-room proton therapy utilizing active scanning PBT.

## Methods

**Figure 1:**
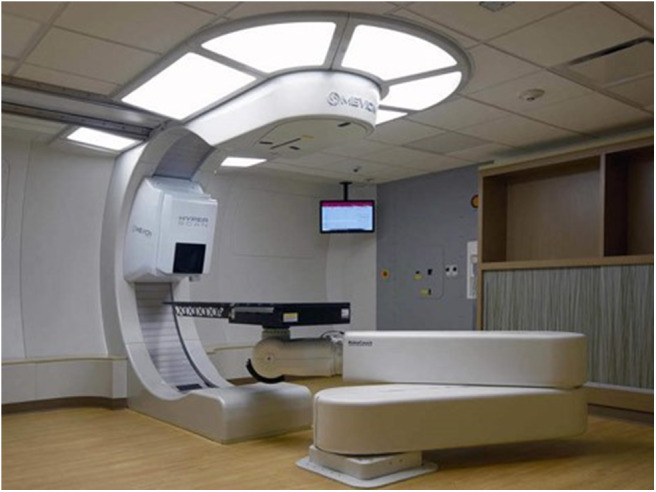
Mevion S250i Proton Therapy System with Hyperscan Pencil Beam Scanning and Adaptive Aperture installed at the MedStar Georgetown University Hospital Proton Center.

The department of radiation medicine at MedStar Georgetown University Hospital is comprised of nine proton accredited attending radiation oncologists servicing our home institution. All physicians completed a training program which included didactic sessions and proctored cases. Additionally, several radiation oncologists received additional experience at outside proton centers. Our facility contains two linear accelerators for standard x-ray treatments, a fully equipped HDR room, two CyberKnife machines, and, most recently, the Mevion S250i proton accelerator with HyperScan pencil beam scanning with Adaptive Aperture (Figure 1). The Mevion S250i proton therapy delivery system is a single gantry system utilizing a synchrocyclotron to deliver a proton beam with energies up to 250 MeV. All patients undergo computed tomography (CT)-based radiation treatment planning simulation (GE LightSpeed RT16). Internal motion is accounted for by utilizing various measures including 4D CT imaging, abdominal compression, and the placement of internal fiducials at the discretion of the treating radiation oncologist. Treatments are delivered utilizing active scanning proton radiotherapy with HyperScan technology up to a maximum depth of 32 cm and field size of 20 cm in diameter. Treatments are optimized utilizing the Adaptive Aperture, a multileaf collimator system designed for active scanning proton therapy to replace conventional proton therapy apertures and can allow for layer-by-layer collimation to create a sharp lateral penumbra to optimize dose delivery to a planning target volume (PTV). Robustness evaluations are then performed upon the clinical target volume (CTV) across a total of 8 scenarios accounting for worst-case perturbations. These include six scenarios to account for setup uncertainties of ± 5 mm in the left-right, superior-inferior, and anterior-posterior directions and two scenarios accounting for range uncertainties of ± 3.5% (34). The system is equipped with a 190° rotating gantry and a robotic couch with six degrees of freedom and 270° couch rotation. Proton beam radiotherapy treatment plans were generated using the RayStation Treatment planning software with Monte Carlo optimization algorithms (RaySearch Americas, Inc. New York, NY). All patients are set-up daily utilizing orthogonal kV imaging to match to bony anatomy with subsequent final adjustment to fiducials, when available. Treatment set-up and delivery is verified with regularly scheduled quality assurance CT scans during treatment.

Patients treated with PBT participated in an institutional review board-approved patient registry (IRB Study# 00001269). Patient demographic data, diagnosis, treatment modality, and geographic referral patterns were obtained for all patients treated with PBT. Anatomical treatment sites and histologic subtypes were determined by retrospective chart review and were categorized based on cancer site, which included the following: central nervous system (CNS), head and neck (H&N), thorax, gastrointestinal (GI), genitourinary (GU), and breast. Pediatric patients under the age of 18 were identified and classified separately. Prior to receiving PBT, all patients were evaluated by a site-specific radiation oncologist. Each case was then discussed in a weekly peer-reviewed PBT conference that evaluated the pertinent clinical history and their suitability for PBT. Institutional suitability criteria for PBT included the following: limiting dose to surrounding OARs, cases of reirradiation, significant medical comorbidities precluding conventional x-ray therapy, such as connective tissue disorders, and pediatric patients.

Treatment plans for patients who received PBT as a portion of their treatments were evaluated. The fractional distribution of PBT vs. x-ray therapy was determined. The rationale for patients receiving a combination of proton and x-ray treatments was obtained by chart review and a detailed discussion with the treating radiation oncologist. Rationale for combination therapy was categorized based on the following factors: proton treatment machine downtime, delay in insurance approval, treatment planning complexity, or preplanned combination therapy.

Systemic therapy data was obtained from the electronic medical record and corroborated with the treating radiation oncologist. Utilization of systemic therapy was classified as follows: concurrent, neoadjuvant, adjuvant, combined neoadjuvant and adjuvant, or none given. Concurrent systemic therapy was defined as any systemic agent delivered during the course of radiation therapy. Neoadjuvant and adjuvant systemic therapy administration was defined as planned systemic therapy given prior to or following completion, respectively, of radiation therapy in the definitive setting. Geographic distance traveled for patients treated with PBT was calculated in miles based on patient registered zip code. Distances were calculated utilizing Google maps and were reported as the minimum distance traveled by car from their registered zip code to the proton center (Alphabet, Inc. Mountain View, CA). Referral patterns for patients were also analyzed for all patients. Classifications for patients included internal referrals for patients referred from within the MedStar hospital network, outside facility referrals encompassing patients referred from physicians outside of the MedStar hospital network, and patients who are self-referred.

Insurance approval data was evaluated and categorized for all patients who were initially denied PBT, and was analyzed using the following: the 2017 American Society for Therapeutic Radiation Oncology (ASTRO) model policy group category, length of time from initial submission to approval, insurance submission to treatment start, and appeal steps taken for insurance approval. The ASTRO group categories are defined in detail in their proton beam therapy model policy (35). As previously published, group 1 consists of sites that frequently support the use of PBT including pediatric cancers, CNS tumors, primary hepatobiliary cancers, head and neck cancers, retroperitoneal sarcomas, and reirradiation cases. Group 2 includes all other disease sites when treated as a part of a clinical trial or multi-institutional patient registry.

## Results

**Figure 2:**
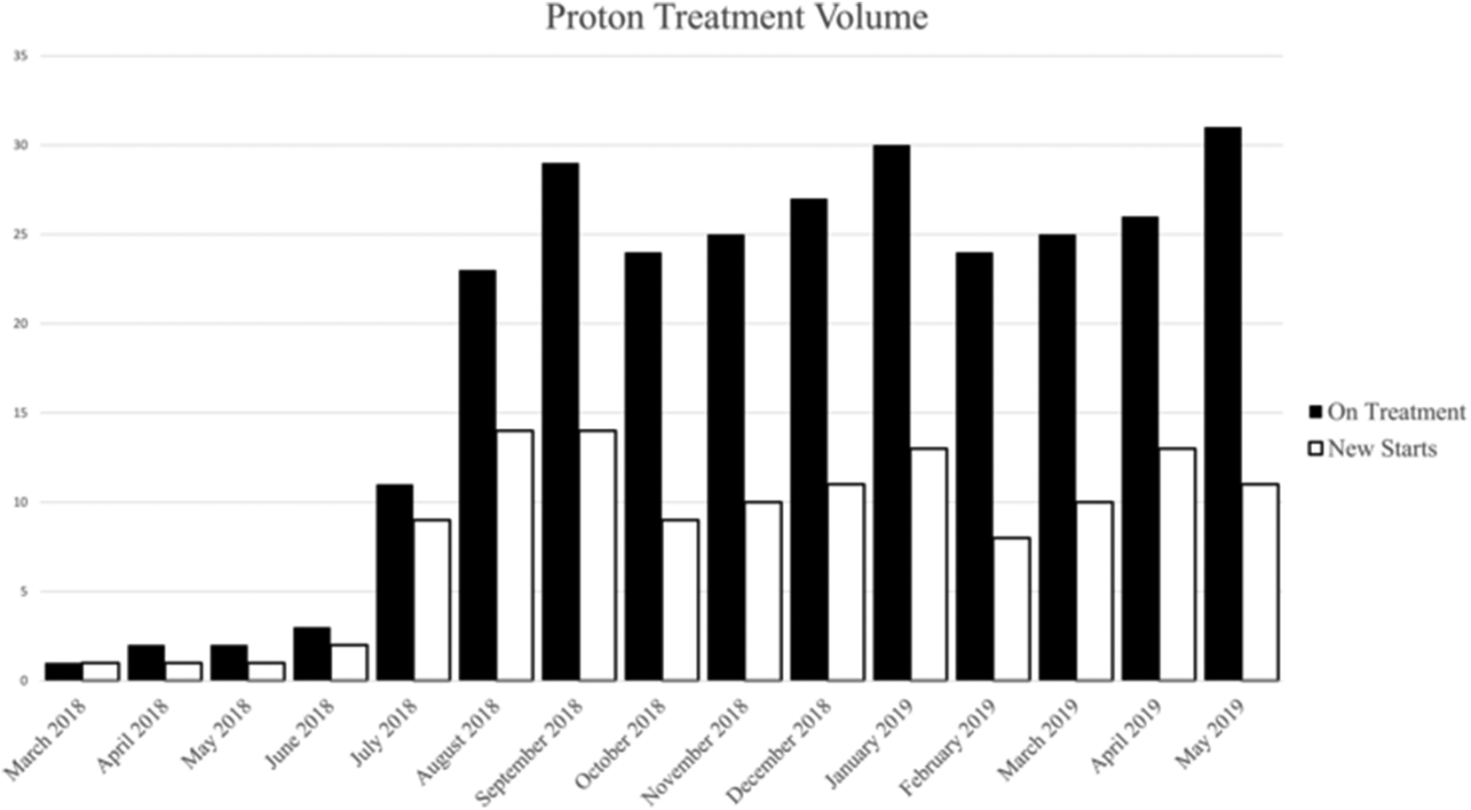
Number of patients on treatment (white) and number of patient new starts (black) by month during the first year.

Between March 2018 and June 2019, 132 patients with a diverse set of disease sites were treated with PBT at the MedStar Georgetown University Hospital Proton Center. An average of 12 patients were treated per day over the total period and a total of 3,285 fractions of PBT were delivered over this period. An average of 8 patients were treated daily during the first 6 months of treatment which increased to an average of 20 patients treated per day by the last 6 months. As shown in Figure 2, the volume of patients treated with PBT increased slowly over the first 4 months as the department built familiarity with the new treatment modality and quickly reached capacity by 6 months. Of the total 132 patients treated, the majority were male (*n* = 73, 55%) and were >65 years (*n* = 73, 55%), similar to the general x-ray treatment population. The majority of patients received chemotherapy as a component of their treatment. Fifty-seven patients (43%) received concurrent chemotherapy during PBT. The rationale for utilizing PBT compared to x-ray radiotherapy was primarily due to limitations of normal tissue tolerance of adjacent OARs (*n* = 88, 67%) and reirradiation cases (*n* = 25, 19%) (Table 1).

**Table 1:** Patient and treatment characteristics.

**Characteristic**	**No. of patients**	**%**
**Sex**		
Male	73	55
Female	59	45
**Age (years)**		
>65	73	55
18–65	54	41
<18	5	4
**Site**		
CNS	29	22
GI	27	20
GU	26	20
Thorax	18	14
H&N	14	11
Breast	10	8
Pediatrics	5	4
Other	3	2
**Rationale for proton**		
Adjacent OAR	88	67
Reirradiation	25	19
Patient comorbidities	15	11
Pediatrics	4	3
**Chemotherapy**		
Concurrent	57	43
None	55	42
Adjuvant	10	8
Neoadjuvant + Adjuvant	9	7
Neoadjuvant	1	1

Of the twenty-five reirradiation cases, the thorax, CNS, and H&N were the leading treatment sites (*n* = 6, 24%; *n* = 6, 24%; *n* = 5, 20% respectively). Reirradiation for CNS malignancies included recurrent glioma (*n* = 2) and ependymoma (*n* = 2). Retreatment for non-small cell lung cancer (NSCLC) comprised the majority of thoracic reirradiation cases (*n* = 5). One pediatric patient was retreated for progressive rhabdomyosarcoma. Furthermore, 28 patients were enrolled in clinical trials. The leading disease sites included GU and thoracic malignancies with 12 GU and 11 thoracic patients participating (Table 2).

**Table 2:** Clinical trial involvement and reirradiation characteristics.

**Characteristic**	**No. of patients**	**%**
**Clinical trial**	28	
GU	12	43
Thorax	11	39
Breast	5	18
**Reirradiation**	25	
Thorax	6	24
CNS	6	24
Head and neck	5	20
GI	3	12
GU	3	12
Other	1	4
Pediatrics	1	4

**Figure 3:**
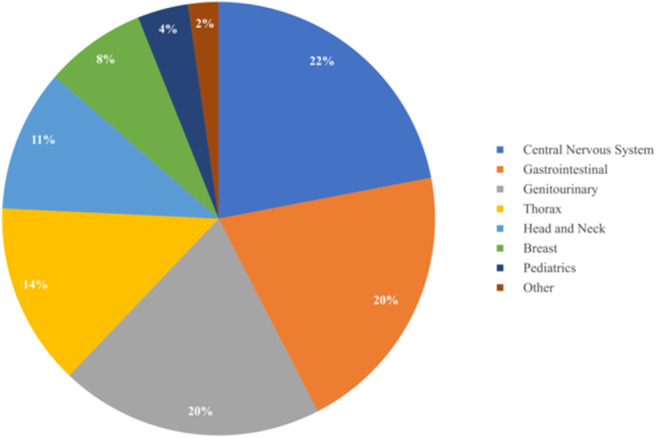
Disease site breakdown of patients receiving PBT.

**Figure 4:**
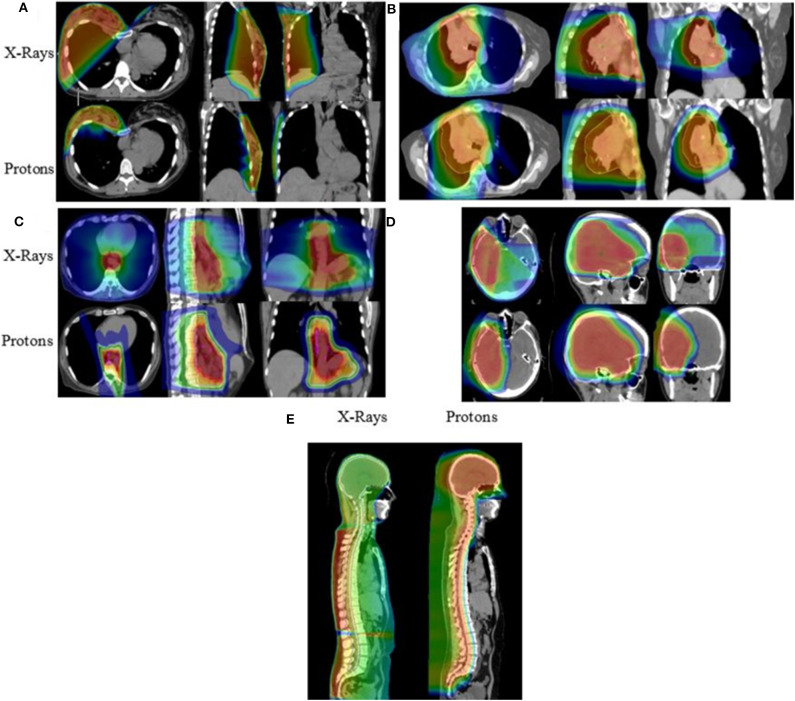
Isodose distributions of the proton radiotherapy plans with comparison x-ray plans generated for patients treated with PBT including patients with **(A)** breast cancer, **(B)** locally advanced NSCLC, **(C)** esophageal cancer, **(D)** primary low-grade glioma, and **(E)** ependymoma receiving craniospinal irradiation.

As summarized in Table 3 and Figure 3, a diverse group of disease sites were treated, with the most common subsites being CNS, GI, or GU malignancies. The primary CNS malignancies treated included gliomas (*n* = 10), followed by meningiomas (*n* = 6), pituitary adenomas (*n* = 4), and ependymomas (*n* = 3). Genitourinary and GI cases followed CNS as the most frequently treated disease sites. Prostate adenocarcinoma was the leading GU malignancy treated (*n* = 24). Of the GI tract, the most common malignancy was esophageal cancer with similar proportions of adenocarcinoma (*n* = 5) and squamous cell carcinoma (*n* = 4). Esophageal cancer was closely followed by cholangiocarcinoma (*n* = 8) and pancreatic adenocarcinoma (*n* = 5). Of the 18 patients treated for thoracic malignancies, the majority of the patients had NSCLC (*n* = 13), adenocarcinoma being the most common histology (*n* = 8). Head and neck cancer patients were treated across a variety of subsites including: oropharynx (*n* = 5), salivary gland (*n* = 4), oral cavity (*n* = 2), and larynx (*n* = 1). In the H&N, the most common histology was squamous cell carcinoma (*n* = 8), followed by adenoid cystic carcinoma (*n* = 3). Ten patients with breast cancer were treated with PBT and seven of the 10 had left sided disease. Finally, five pediatric cases treated included: Ewing's sarcoma (*n* = 2), rhabdomyosarcoma (*n* = 2), and a high-grade peripheral nerve sheath tumor (*n* = 1). Representative PBT treatment plans generated in the RayStation treatment planning software for different disease sites alongside comparison x-ray treatment plans are shown in Figure 4.

**Table 3:** Disease site breakdown.

**Characteristic**	**No. of patients**	**%**
**Central nervous system**	29	22
Histological subtype		
Low-grade glioma	7	24
Meningioma	6	21
Pituitary adenoma	4	14
High-grade glioma	3	10
Ependymoma	3	10
Chordoma	1	3
Craniopharyngioma	1	3
Other	4	14
**Gastrointestinal**	27	20
Subsites		
Esophagus	10	37
Cholangiocarcinoma	8	30
Pancreas	6	22
Anus	2	7
Hepatocellular carcinoma	1	4
**Genitourinary**	26	20
Histological subtype		
Prostate adenocarcinoma	24	92
Urothelial carcinoma	1	4
Urethral carcinoma	1	4
**Thorax**	18	14
Histologic subtype		
NSCLC	13	72
Thymoma	4	22
Lymphoma	1	6
**Head and Neck**	14	11
Subsites		
Oropharynx	5	36
Salivary gland	4	29
Oral Cavity	2	14
Larynx	1	7
Other	2	14
**Breast**	10	8
Histological subtype		
Invasive ductal carcinoma	6	60
Ductal carcinoma *in situ*	3	30
Invasive lobular carcinoma	1	10
Laterality		
Left	7	70
Right	3	30
**Pediatrics**	5	4
Histological subtype		
Ewing's sarcoma	2	40
Rhabdomyosarcoma	2	40
Malignant peripheral nerve sheath tumor	1	20
**Other**	3	2
Histological subtype		
Liposarcoma	1	33
Other	2	67

Of the total 132 patients, 82 patients (62%) were treated with PBT as a single modality, and 50 patients (38%) received both proton and at least one fraction of x-ray therapy. Of the patients who received combined modality treatments, the most common rationales included a planned course of mixed modality treatment (*n* = 18, 36%) and unplanned proton machine downtime (*n* = 17, 34%). Patients who received a mixture of radiotherapy modalities due to unplanned machine downtime received a median of three x-ray treatment fractions (Table 4).

**Table 4:** Plan and insurance characteristics.

**Characteristic**	**No. of patients**	**%**
**Plan**		
100% PBT	82	62
75–99% PBT	30	23
50–74% PBT	5	4
<50% PBT	15	11
**Reason for mixture**		
Preplanned combination therapy	18	36
Unplanned machine downtime	17	34
Planned machine downtime	7	14
Proton treatment planning complexity	6	12
Insurance delay	2	4
**Unplanned machine downtime**		
>3 days	52	39
2–3 days	29	22
1 day	18	14
0 days	33	25
**Initial proton denial**		
Yes	27	20
No	105	80
**Measures taken for insurance approval**		
Peer-to-peer conducted	17	63
Secondary insurance approved	3	11
Approved upon second appeal	3	11
Photon and proton comparison plan required	2	7
Insurance refused to cover fully	8	30
**2017 ASTRO Model Policy Group**		
Group 1	12	44
Days to insurance approval from submission (median)	17	
Days to simulation from submission (median)	22	

Of those patients who were ultimately treated with PBT, 27 patients (20%) were initially denied insurance coverage. Of these denials, approximately half (*n* = 12, 44%) met ASTRO guidelines for group 1 PBT suitability. A peer-to-peer option with their primary radiation oncologist was required for insurance approval in 17 patients (65%). Three patients (12%) required an additional written appeal prior to treatment approval and two patients (8%) required a comparison plan of proton vs. x-ray treatments prior to approval. Of note, three patients (12%) used a secondary insurance to obtain insurance approval. Insurance was ultimately denied for PBT for eight patients. The appeal process for patients who were denied PBT required a median of 17 days (range, 4–88 days) from initial insurance submission to approval. As a consequence, initial simulations for these patients occurred at a median of 22 days (range, 7–119 days) from initial insurance submission (Table 4). Importantly, many patients who were initially denied PBT were ultimately not treated with PBT and are not reflected in the aforementioned data.

From a geographic standpoint, the majority of patients traveled from within the District of Columbia and the surrounding states, Maryland and Virginia (*n* = 126, 95%), with a median distance traveled of 14.4 miles. Patients were also most likely to be internally referred for treatment (*n* = 76, 58%), followed by outside referrals (*n* = 40, 30%). Seventy-four patients traveled between 5 to 20 miles (56%) and forty-three patients traveled more than 20 miles (33%). Finally, three patients traveled from out of state and three patients traveled internationally (Table 5).

**Table 5:** Geographic details.

**Characteristic**	**No. of patients**	**%**
**Distance traveled (miles)**		
<5	12	9
5–10	32	24
10–20	42	32
>20	43	33
International	3	2
**Total miles traveled domestically**	3,446	
Average	26.7	
Median	14.4	
Range	1.3–431	
**Referral**		
Internal	76	58
Outside	40	30
Self	15	12

## Conclusions

The present study sought to characterize the initial experience of a single-room integrated proton therapy center opened within an existing comprehensive cancer center at an academic teaching hospital in a major metropolitan area with particular focus on the spectrum of disease sites treated, the utilization of systemic therapies, the geographic reach, and the impact of insurance approval on treatment times.

From a geographic standpoint, the installation of the proton center drew in patients largely from the surrounding areas within Washington, DC, Virginia, and Maryland, although a small number of patients traveled from out of state or internationally for treatment. This increase in geographic outreach allowed the facility to reach treatment capacity within 6 months of operation.

A surprisingly diverse group of disease sites were treated during the first 16 months of operation. Tumors of the central nervous system were the most common malignancy treated, primarily comprised of gliomas, followed by meningiomas and pituitary adenomas. This was largely driven by the need to reduce doses to the surrounding normal brain and critical OARs, such as the optic apparatus and brainstem, to minimize late effects of radiation treatments, including neurocognitive decline (24, 36). Moreover, primary malignancies of the CNS constituted the largest proportion of the reirradiation cases treated at our institution. Reirradiation of the brain is uniquely challenging given patients have often previously undergone surgery, chemotherapy and high dose radiation therapy, and are thus more susceptible to tissue damage (24, 37).

For decades, proton centers have focused on treatment of tumors of the CNS due to its inherent lack of motion, tissue homogeneity, and reproducible setup. At our institution, we wanted to expand into other, more challenging sites including the thorax and gastrointestinal tract, in order to explore more novel uses of the available technology. As such, one-third of all patients treated were diagnosed with either thoracic or GI malignancies. Proton beam radiotherapy was utilized primarily for definitive treatment of esophageal cancer and thoracic malignancies to minimize dose delivered to the lungs, heart, and spinal cord. This was especially important in patients undergoing reirradiation where PBT was utilized to better spare the previously irradiated spinal cord. The ability of PBT to decrease heart dose and spare various cardiac substructures has been well-established in numerous dosimetric studies and, clinically, increasing heart doses have been shown to have a negative impact on overall survival (38–41). The physical characteristics of PBT providing additional normal tissue sparing also allowed for dose escalation within the liver for treatment of primary intrahepatic malignancies, which has demonstrated high rates of local control in patients with both hepatocellular carcinoma and intrahepatic cholangiocarcinomas (26). Patients treated with PBT for thymomas have also been shown to have excellent outcomes with significant dosimetric advantages in reducing doses to surrounding critical structures which have been shown to decrease rates of both early and late toxicities (42, 43). This can be especially important given the younger age of patients diagnosed with thymomas and the long natural history of the disease in treated patients.

Incorporation of particle therapy at our pre-existing NCI designated comprehensive cancer center was critical to allow access to our multidisciplinary team, including medical oncology, surgical oncology and advanced inpatient care. Half of our patients were able to be treated with chemotherapy during PBT treatments without the logistical challenges of transportation to the main academic center. Access to advanced oncologic multidisciplinary teams allowed for seamless integration of PBT into standard of care practices as well as clinical trial enrollment for patients at the same cancer center (20, 44, 45). The addition of PBT at the main campus also allowed for easier and cost-effective integration of the existing simulator and radiation clinical staff, which enabled uninterrupted oncologic care with transitions between x-ray and proton therapies for planned combined modality therapy or machine maintenance.

One significant challenge continues to be insurance coverage for PBT. Initially published in 2014 and recently updated and expanded in 2017, ASTRO reported a model policy list of expert recommended indications for insurance coverage of PBT (35). However, insurers have yet to fully implement these expert consensus recommendations. Twenty percent of our patients ultimately treated with PBT were initially denied for insurance coverage despite half of those patients qualifying for ASTRO model policy group 1. These insurance denials resulted in significant resource cost to the department, including numerous physician peer-to-peer discussions, advocacy letters, and comparison x-ray vs. proton plans for treatment rationalization. More importantly, the insurance denials resulted in significant delays in patient care with most patients delayed by at least 1 month prior to their first treatment. Our results are in line with recently published experiences from MD Anderson where a significant number of patients were denied PBT coverage by private insurance with a subsequent approval process that required a similar significant time and resource investment from the radiation department (46). The appeal process, as demonstrated by other institutions, also leads to significant delays to the start of proton treatment, with adult patients waiting an average of 1 month (46, 47). Treatment delays have been previously demonstrated across various subsites to be detrimental to patient outcomes, including for gynecologic cancers and tumors of the head and neck (48–50). Another potential complication from extensive treatment delays is increased psychological stress to patients resulting in increased anxiety and depression, which has been anecdotally seen at our institution. These delays may reduce patient compliance with prescribed treatments and adversely impact their treatment outcomes (51, 52).

Limitations of the present study include its retrospective nature and the fact that the analysis only included patients that ultimately received PBT. There may have been patients who would have benefitted from PBT but were denied treatment by insurance or not pursued due to expected insurance denial and subsequent treatment delays. Further investigation into insurance denial patterns and approval rates across all patients is warranted. Additionally, this was a single-institutional analysis in a metropolitan area, which may limit the generalizability of our experience to other centers. Nevertheless, our results mirror other published experiences which suggests that there may be some general applicability to other centers across the country (47, 53).

In conclusion, a single room active scanning proton therapy treatment center provides a viable option for institutions preparing to invest in particle therapy. Incorporation of PBT into an established cancer center allowed for seamless integration of particle therapy into the multidisciplinary oncologic treatments of patients. Following installation of a single room proton center, 132 patients were treated during the first 16 months of operation spanning a wide variety of disease sites, most commonly tumors of the central nervous system, gastrointestinal tract, and genitourinary tracts. Insurance approval for PBT continues to be a resource and time intensive process for patients and providers, and improvements are needed to provide more timely access to necessary cancer care.

## Data Availability Statement

The datasets used and/or analyzed during the current study are available from the corresponding author, without undue reservation, to any qualified researcher.

## Ethics Statement

The studies involving human participants were reviewed and approved by the Georgetown Institutional Review Board and was approved under IRB Study# 00001269. The patients/participants legal guardian/next of kin provided written informed consent to participate in this study.

## Author Contributions

BC, SC, JL, MF, AD, PA, EB, WH, NP, KU, DP, and SR contributed to study concept, design, and/or acquisition of data. BC, JL, MF, and EB completed the data collection. JL, MF, and EB contributed to the data analysis. MF and JL were responsible for drafting the manuscript. All authors contributed to revising and giving final approval to the manuscript. All authors agree to be accountable for all aspects of the work including its accuracy and integrity.

## Conflict of Interest

BC, SC, and JL are paid speakers for Accuray. BC, JL and DP are paid speakers for Mevion. The remaining authors declare that the research was conducted in the absence of any commercial or financial relationships that could be construed as a potential conflict of interest.
